# A Novel Approach Using Serious Game Data to Predict the WISC-V Processing Speed Index in Children With Attention-Deficit/Hyperactivity Disorder: Machine Learning Study

**DOI:** 10.2196/73408

**Published:** 2025-10-14

**Authors:** Jun-Su Kim, Yoo Joo Jeong, Seung-Jae Kim, Su Jin Jun, Jin-Yeop Park, Hyang-Sook Hoe, Jeong-Heon Song

**Affiliations:** 1 AI-based Neurodevelopmental Diseases Digital Therapeutics Group, Korea Brain Research Institute (KBRI), 61, Cheomdan-ro, Daegu, 41062, Republic of Korea; 2 Neurodegenerative Disease Group, Korea Brain Research Institute (KBRI), Daegu, Republic of Korea; 3 Department of Brain and Cognitive Sciences, Gyeongbuk Institute of Science & Technology, Daegu, Republic of Korea

**Keywords:** attention deficit hyperactivity disorder, ADHD, prediction, machine learning, symptom tracking, processing speed, Serious games, digital therapeutics

## Abstract

**Background:**

The processing speed index (PSI) of the Korean Wechsler Intelligence Scale for Children-Fifth Edition (K-WISC-V) is highly correlated with symptoms of attention-deficit/hyperactivity disorder (ADHD) and is an important indicator of cognitive function. However, restrictions on the frequency of testing prevent short-term PSI assessments. An accessible, objective technique for predicting PSI scores would enable better short-term monitoring and intervention for children with ADHD.

**Objective:**

To enable objective and accessible monitoring of cognitive function beyond traditional clinical assessments, this study aimed to develop a machine learning model that predicts the PSI scores of children with ADHD using behavioral data from serious games.

**Methods:**

Sixty-eight children (6-13 y of age) with ADHD were recruited, and after excluding incomplete data, 59 participants were included in the final analysis. The participants completed an initial PSI assessment using the K-WISC-V followed by 25 minutes of engagement with serious game content. Data from the game sessions were used to train machine learning models, and the models’ performance in predicting PSI scores was evaluated using the root mean squared error (RMSE), mean absolute error (MAE), and mean absolute percent error (MAPE), with K-fold cross-validation (k=4) applied to ensure robustness.

**Results:**

Among the individual machine learning models, support vector regression (SVR) had the best performance, with the lowest RMSE of 11.288, MAE of 7.874, and MAPE of 7.375%. The best overall performance was achieved by the ensemble integrating AdaBoost, Elastic Net, and SVR, which recorded the lowest RMSE of 10.072, MAE of 6.798, and MAPE of 6.611%. The predictive accuracy of this ensemble model was highest for PSI scores near the mean value of 100, demonstrating its reliability for clinical applications.

**Conclusions:**

The developed PSI prediction model has the potential to serve as an objective and accessible tool for monitoring cognitive function in children with ADHD. As a complement to traditional assessments, this approach allows continuous tracking of symptom changes and can support more personalized treatment planning in both clinical and everyday settings, which may improve accessibility and adherence. However, the findings need to be validated in larger, more diverse populations, and the long-term feasibility of using serious games in clinical and educational settings must be further examined.

## Introduction

Attention-deficit/hyperactivity disorder (ADHD) is a neurodevelopmental disorder that progresses throughout childhood and continues into adulthood [1]. Accurate assessment and management are essential for determining the best time to intervene and improve treatment outcomes, but the diagnosis of ADHD and other neurodevelopmental disorders often relies on subjective clinical evaluations, which can result in inconsistent or inaccurate diagnoses [2-4]. Additionally, symptom evaluation outside clinical environments, such as at home or school, typically depends on observation rather than objective measurements [5]. Processing speed (PS), that is, the ability to respond to a given stimulus within a limited time frame, may offer valuable insights for treatment and monitoring [6]. Individuals with ADHD exhibit slower PS, which has been shown to hinder an individual’s ability to process and respond to information efficiently, negatively affecting academic performance, adaptability, anxiety levels, and social competence [7,8]. Lower scores on PS measures, such as the processing speed index (PSI), are correlated with greater severity of attention deficits [9].

The PSI concept was first introduced in the Third Edition of the Wechsler Intelligence Scale for Children (WISC-III), and a formal composite score was established in the Fourth Edition (WISC-IV) [10,11]. The WISC is one of the most widely used and highly validated tools for assessing cognitive function in children, making its index scores highly reliable [12-14]. The latest version, WISC-V, includes the PSI as one of its indices and is frequently used with children in both clinical and educational settings [12]. Due to its strong psychometric properties, the PSI is considered a reliable indicator of PS. Beyond its utility in diagnosis, the PSI is also associated with daily functioning and provides a useful way to quantify ADHD symptoms, highlighting its importance in managing ADHD. However, PSI assessment has two key limitations. First, for effective PSI measurement, trained professionals, such as clinical psychologists or physicians, must administer and interpret specialized tests. Second, repeated testing can lead to practice effects, which may reduce data validity [15-17]. Consequently, the recommended usage interval for WISC is 1-to 2.6 years, making it inappropriate for continuous monitoring [18-20].

To overcome the methodological limitations of measuring the PSI, this study aims to develop a machine learning model that predicts PSI scores in children with ADHD. The model uses behavioral characteristic data collected from Neuro-World, an artificial intelligence-based serious game classified as Software as a Medical Device (SaMD). Serious games and other digital therapeutics (DTx) have emerged as promising tools for treating neurodevelopmental disorders like ADHD [21-24]. DTx allows continuous, location-independent monitoring, improving adherence and enabling real-time tracking [25,26]. These advantages of DTx may also be useful for cognitive assessment. For example, a recent study demonstrated the feasibility of using mobile platforms for assessing PS and working memory via momentary assessment methods [27]. Building on this, large-scale population studies have used mobile cognitive games to evaluate PS across different age groups and cognitive domains [28]. Although digital tools have shown promise for assessing PS through mobile technologies, most have not been validated against established neuropsychological indices such as the PSI. Moreover, while some research has used machine learning models to predict mild cognitive impairment (MCI) based on Mini-Mental State Examination (MMSE) scores, no study has specifically focused on predicting the PSI from behavioral data [29,30]. In addition, preliminary studies have primarily focused on detecting age-related differences or demonstrating feasibility rather than predicting clinically meaningful cognitive metrics.

Based on the well-established association between ADHD severity and a lower PSI [8], as well as prior evidence of improvements in WISC-V scores following Neuro-World training [31], this study aimed to monitor and track cognitive function related to PS. A systematic evaluation of multiple machine learning algorithms identified the model that exhibited the highest level of performance while maintaining the accuracy and reliability required for PSI prediction from game-derived behavioral data.

## Methods

### Participants

This study was conducted with pediatric participants aged 6-13 years with a diagnosis of ADHD. A total of 34 children with ADHD were recruited between December 2021 and December 2022 (IRB no. 2021-10-080), and an additional 34 children diagnosed with ADHD were recruited between December 2022 and March 2023 (CRIS Registration no. KCT0009326). However, 4 participants were excluded as they refused to participate. The total sample size of the study was 64 participants: 51 (79.7%) male and 13 (20.3%) female participants. The average age of the participants was 8.5 (SD 1.7) years. The participants were all South Korean and were recruited from a single clinical institute. In this study, demographic factors such as socioeconomic status and the education level of the legal guardian were not collected.

### Ethical Considerations

This study was approved by two different institutional review boards (IRBs) due to the different recruitment phases. The initial phase of the study was reviewed and approved by the IRB of Keimyung University Dongsan Hospital (IRB no. 2021-10-080), which is certified by the Korea Laboratory Accreditation Scheme (KOLAS), a nationally recognized accreditation body in South Korea. The subsequent phase of the study was registered with the Korea Clinical Research Information Service (CRIS Registration no. KCT0009326), which is accredited by the World Health Organization (WHO). Participants were recruited from the Department of Psychiatry at Keimyung University Dongsan Hospital. Before study enrollment, written informed consent was obtained from the legal guardians of all participants, and the study procedures were explained to the participants to secure their assent. The consent form specified that the collected data could be used not only for the primary study but also for future research and secondary analyses without requiring additional consent. All collected data were deidentified and anonymized to ensure confidentiality. As compensation, participants were provided with coverage for transportation costs, and their legal guardians received professional psychological assessment results and evaluation reports. No identifiable images of participants are included in this manuscript or supplementary materials, and no personal information was disclosed.

### Experimental Methodology

**Figure 1.**
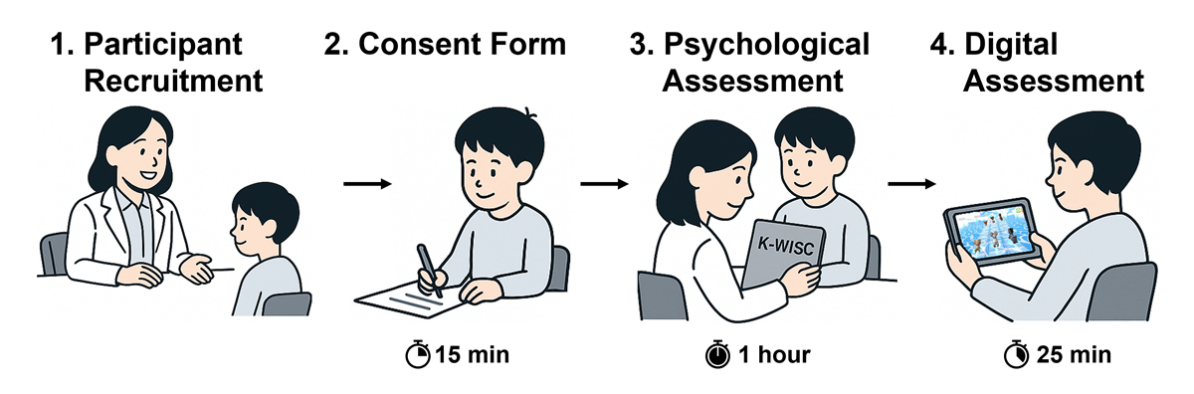
Clinical trial process. A total of 64 children diagnosed with ADHD participated in this study. After completing consent forms, the participants underwent a 1-hour psychological assessment using K-WISC-V. After the K-WISC-V assessment, the children performed a digital assessment using Neuro-World software for 25 minutes. Artificial intelligence–generated image (Generator: ChatGPT, OpenAI; July 14, 2025; Requestor: Jun-Su Kim). ADHD: attention-deficit/hyperactivity disorder; K-WISC-V: Korean Wechsler Intelligence Scale for Children-Fifth Edition.

To minimize disruptions to routine that might affect performance on attention-based activities, we requested that the participants follow their usual daily routines and take their prescribed medications as scheduled. After the participants completed consent forms, they underwent a psychological assessment using the K-WISC-V, which took 1 hour. The participants were provided with a 10-minute break after the psychological assessment and performed gameplay with the developed serious game content for 25 minutes. The overall clinical trial process is presented in Figure 1.

To ensure an objective setting for gameplay, all participants performed the serious game content independently in a quiet environment free from external distractions and noise. To ensure participant comfort, a pause button was provided, which allowed participants to stop if they experienced dizziness or required a break.

### K-WISC-V: Processing Speed Index (PSI)

WISC is widely recognized as a comprehensive instrument for assessing cognitive functioning in children [31]. The WISC-V has been translated and culturally adapted for use in Korea, and this version is known as the Korean Wechsler Intelligence Scale for Children-Fifth Edition (K-WISC-V). The K-WISC-V is targeted to children and adolescents (aged 6 to 16 years and 11 mo) and includes core indices for the verbal comprehension index (VCI), perceptual reasoning index (PRI), working memory index (WMI), and PSI [32,33]. Each assessment is conducted individually, with scores calculated based on established criteria and then converted to age- and gender-specific scaled scores. The index scores are standardized with a mean of 100 and an SD of 15, enabling comparisons of an individual’s abilities with those in the same age group [34].

PSI, a critical component of the K-WISC-V, assesses visual PS and psychomotor efficiency by measuring reaction time, spatial navigation, discrimination, and accuracy. Attention and hand-eye coordination are the major contributing factors to the PSI [35]. The PSI captures learning efficiency, attentional control, and executive functioning, which are related to various cognitive dimensions in children. Higher PSI scores indicate faster and more accurate visual decision-making, reflecting greater efficiency in time management for learning tasks and assessments [36]. Symbol search and coding, the two core subtests of PSI, are described in Table 1.

**Table 1.** Detailed description of core PSI subtests.

Subtest	Description
Symbol Search	A target symbol is given to the participants, who are asked to identify and mark the matching symbols in a given set within a limited time. This task assesses visual search speed and coordination-discrimination ability.
Coding	A set of numbers is matched with symbols, and participants are required to write down the symbols corresponding to a given set of numbers within a limited time. This task evaluates visuomotor coordination, associative learning ability, and visual short-term memory.

### Serious Game Contents for PS Measurement

PS refers to the ability to quickly complete simple tasks while maintaining focus. Cognitive function assessments typically use a brief neuropsychological task that requires approximately 3-5 minutes [37], but neuropsychological tasks used to evaluate sustained attention generally require continuous engagement for 15 to 20 minutes [37]. Based on these observations, we designed serious game sessions with a total duration of 25 minutes. Several recent studies have found that interventions using the software Neuro-World, developed by Woorisoft Co, Ltd (Republic of Korea), improve decision-making ability, overall cognitive PS, working memory, and ADHD-related symptoms, including attention deficit, hyperactivity, and internalization and externalization disorder, in children with ADHD [38-40]. Building on these findings, Neuro-World was used in this study to monitor and track cognitive function related to PS in pediatric participants diagnosed with ADHD.

**Figure 2.**
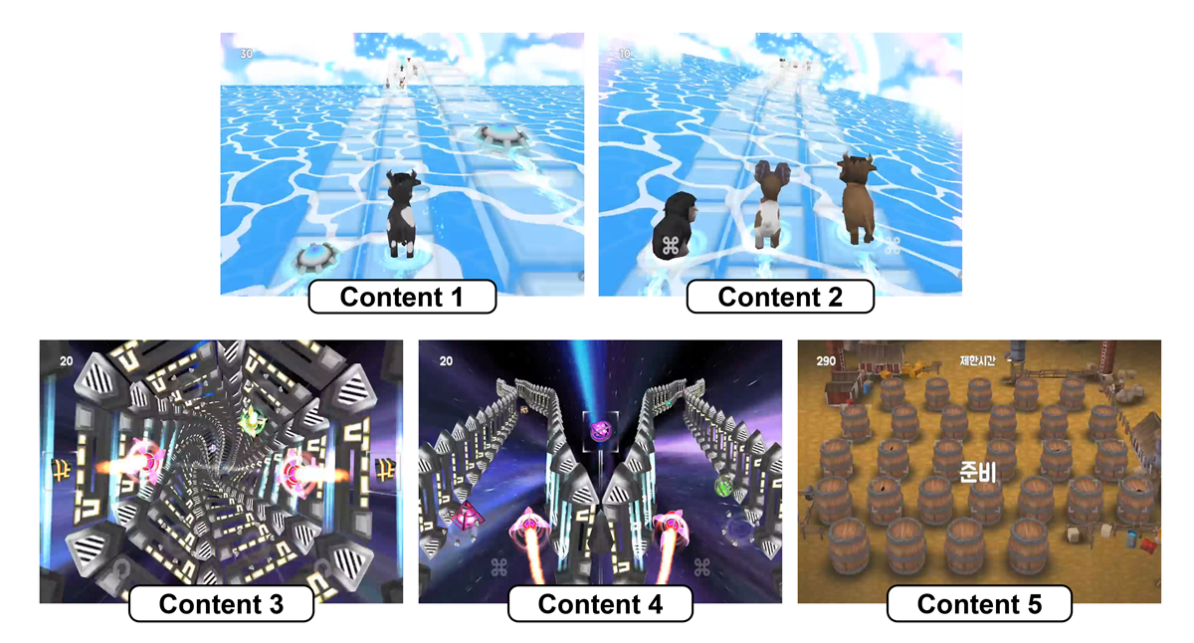
Five Neuro-World serious game content modules used to evaluate processing speed. Content 1: identifying and moving animals; content 2: rearranging sequences; content 3: avoiding obstacles; content 4: dual obstacle avoidance; and content 5: memory matching.

As shown in Figure 2, Neuro-World comprises 5 independent serious game content modules. Each module takes approximately 5 minutes to complete; thus, the whole digital assessment procedure involves a total of 25 minutes of engagement. The structure of the assessment was designed to assess both cognitive performance and sustained attention within a single session. The sequence of content modules was randomized, and the next content module began automatically after completion of the previous content module. To address potential device or platform bias, all participants completed the game-based assessments using standardized Android tablets (≥9.7-inch screens) with identical touch screen interfaces. No external input devices were used. Table 2 summarizes the technical specifications of the devices and software.

**Table 2.** Device and software specifications used in the Neuro-World-based digital assessment.

Component	Specification
Item name	Cognitive Therapy Software
Model name	Neuro-World DTx-ADHD
Operating system	Android
Device type	Tablet (9.7 inches or larger)
Input method	Touch screen

### Behavioral Feature Extraction From the Serious Game

The following methodologies were used to extract behavioral characteristics from the 5 distinct content modules. First, the complexity of each content module was dynamically adjusted based on the participant’s performance:

- Play speed: As the difficulty level increased, the speed of content progression accelerated, intensifying the sense of urgency during task performance.
- Number of items to remember: The cognitive load was gradually increased by requiring the participants to memorize a larger number of items.

Previous studies have shown that the variability of response time is strongly correlated with ADHD [41-43]. Based on these observations, the game content was designed to gradually reduce the effective time available for problem-solving as the difficulty increased, requiring participants to process information more quickly and efficiently.

Second, a series of data were collected during the cognitive assessment using the Neuro-World content modules: 𝑥1, total number of trials conducted in one content module; 𝑥2, accuracy rate; 𝑥3, total score; 𝑥4, total number of screen touches; 𝑥5, unnecessary actions during content module performance, calculated as the difference between the total number of screen touches and the number of touches required for correct answers; 𝑥6, highest level of performance; 𝑥7, average level of performance; 𝑥8, SD of performance level; 𝑥9 and 𝑥10, average time to touch the screen after a task was presented (ie, response time) and its SD; 𝑥11 and 𝑥12, average time to the final touch required to complete each game (ie, task completion consistency) and its SD; and 𝑥13 and 𝑥14, average decision-making time, calculated as the difference between the final and first touch times and its SD.

Exploratory studies have suggested that individuals with ADHD exhibit deficits in sustained attention [44-47]. Based on these findings, we divided the total content performance into 2 phases and aimed to quantitatively assess whether the participants’ performance improved over time or deteriorated due to reduced concentration, thereby capturing changes in their cognitive task-solving processes. The 14 variables were recorded separately in the 2 phases: 𝑥1:14 (initial phase) and 𝑥15:28 (later phase). The 28 features were extracted from each of the 5 content modules, resulting in a total of 140 variables (𝑥1:140) [48].

### Data Preprocessing

Machine learning models, including support vector regression (SVR), random forest, and AdaBoost, generally require a sample size that is at least 10- 20 times larger than the number of input features to ensure stable learning performance [49]. In this study, the initial features consisted of 140 variables extracted from serious game performance data. To minimize the risk of overfitting and enhance the stability of the prediction model, dimensionality reduction was required. Accordingly, to extract the appropriate variables from the data to enhance the model’s predictive performance, the correlation-based feature selection (CFS) method was applied. CFS is a filter-based method that identifies an optimal set of variables by maximizing the correlations of the variables with the PSI while minimizing correlations between variables [50]. The effectiveness of the selected variable set in CFS is measured using <Equation 1>. In this equation, *Merits* represent the cumulative correlation between the variables and the external variable; *k* denotes the number of variables; $\overset{-}{r_{zi}}$ represents the average correlation between variables and the external variable; and $\overset{-}{r_{ii}}$ denotes the average intercorrelation between variables [51-53]. The value of *Merits* corresponds to the usefulness of the variable set for prediction; a higher value indicates a stronger correlation with the target class and reduced redundancy among variables. A higher value of *Merits* therefore signifies not only that the variable set is more closely aligned with the target class but also that it contributes to improved model performance by reducing irrelevant or redundant information.


(1) $$Merits=\frac{k\overset{-}{r_{zi}}}{\sqrt{k+k\left(k-1\right)\overset{-}{r_{ii}}}}$$

Using the CFS method, 6 variables were selected from 140 content variables. In addition, the variables gender and age were included, resulting in a total of 8 variables used as inputs for the machine learning models in the analysis. The selected variables are listed in Table 3.

**Table 3.** List of input variables for the machine learning model.

Index	Variable (𝑥n)	Descriptions
1	Con1_answer_rate_back (𝑥16)	Accuracy rate in the later phase of content 1
2	Con2_touch_count_diff_back (𝑥47)	Number of unnecessary touches in the later phase of content 2
3	Con3_user_touch_count_back (𝑥74)	Total number of touches in the later phase of content 3
4	Con5_total_score_front (𝑥115)	Total score in the initial phase of content 5
5	Con3_first_touch_time_mean_front (𝑥65)	Mean response time to the first touch in the initial phase of content 3
6	Con1_decision_time_mean_front (𝑥13)	Mean decision-making time in the initial phase of content 1
7	Gender	Participant’s gender
8	Age	Participant’s age

### Machine Learning Models Used for Predicting the PSI

To predict the PSI scores of children diagnosed with ADHD, we collected performance data from serious games, trained various machine learning models using the selected variables, and compared the predictive performance of the models. We utilized 4 different machine learning models: random forest, AdaBoost, SVR, and Elastic Net. The characteristics of each model are described below.

Random forest is an algorithm that enhances prediction performance by ensemble learning with multiple decision trees [54]. The random forest algorithm constructs regression trees, processes an input vector composed of feature values corresponding to specific training instances, and averages the predicted outputs from each tree [55]. After creating several training data subsets, a bagging method is applied to reduce correlations among different decision trees to reduce variance [56]. This bagging characteristic enhances the predictive accuracy and stability of the random forest algorithm [54].

AdaBoost (Adaptive Boosting) is a boosting algorithm that iteratively trains a series of weak learners and combines them to form a robust ensemble model [57]. Unlike other boosting methods, AdaBoost applies the relative instead of absolute error, giving more weight to examples with lower values [58]. Additionally, AdaBoost continues to run even if the error rate exceeds 0.5 and supports user-defined iterations, allowing performance improvement over initial iterations [58].

SVR solves regression problems by extending binary classification through convex optimization formulations [59]. SVR generalizes support vector machines to adapt to regression problems and introduces an ε-insensitive region (tube) to reformulate the optimization problem [60]. Unlike other multivariate regression models, support vector machines can learn nonlinear functions with the kernel trick, which maps independent variables in a higher dimensional feature space [61]. The ε-insensitive region approximates continuous functions well and balances model complexity with prediction errors [60].

Elastic Net, proposed by Zou and Hastie in 2005, was developed to estimate the regression coefficient ß in situations where p>n [62]. Elastic Net combines the concepts of Lasso and Ridge Regression to estimate ß [63]. The l2-norm penalty tends to reduce coefficients toward zero while retaining all predictors in the Elastic Net model, potentially leading to multicollinearity, whereas the l1-norm penalty automatically selects key information and continuously reduces redundant information [64]. By combining both penalties, Elastic Net effectively mitigates multicollinearity and enables the group selection of correlated features [65,66].

### Machine Learning Model Training and Hyperparameter Optimization for PSI Prediction

To develop an optimal PSI prediction model, we trained the machine learning model and specifically optimized the hyperparameters of each model. No power analysis was conducted because of the exploratory nature of the study, but the use of dimensionality reduction and cross-validation mitigated overfitting and supported generalizability. To prevent issues such as limited generalizability due to the small sample size, we implemented a robust machine learning framework incorporating dimensionality reduction, k-fold cross-validation, hyperparameter tuning via grid search, and random shuffling to mitigate overfitting. Of the initial dataset comprising 64 participants, 5 were excluded because of missing data. Thus, 59 datasets were available for analysis, divided into a training set of 47 (80%) and an independent test set of 12 (20%).

Given the relatively small sample size of this machine learning algorithm study, we also used k-fold cross-validation to ensure robust model validation. The number of folds (k) can be flexibly determined based on the dataset size and the model’s structural complexity and inherent characteristics [67-69]. Following this rationale, we experimented with different k values (k=3, 4, 5, and 6) and chose k=4 to achieve a balanced distribution among the training, validation, and test sets. In each fold, approximately 35-36 samples were used for training and 11-12 samples were used for validation. This configuration ensured that the size of the validation set closely matched the final test set, allowing for more effective parameter tuning under conditions similar to final testing.

Generalizability was further enhanced by performing randomized shuffling in each fold (shuffle=True), ensuring that the final test set remained completely unseen throughout training and tuning. A grid search was performed within each fold to optimize hyperparameters, and the optimal settings for each model are presented in Table 4. For example, the random forest model achieved the best performance with “max_features=log2”, “n_estimators=50”, “max_depth=10”, and “min_samples_split=2”.

**Table 4.** Hyperparameter grids and selected optimal values for each machine learning model.

Model and hyperparameter		Grid search range	Optimal value
Random forest			
	max_features	[’sqrt’, 'log2']	log2
	n_estimators	[50, 100, 200, 300]	50
	max_depth	[3,5,7,10]	10
	min_samples_split	[2-5]	2
AdaBoost			
	n_estimators	[50, 100, 200, 300]	200
	max_depth	[3-10]	5
	learning_rate	[0.01, 0.05, 0.1, 0.5, 1.0]	1.0
Elastic Net			
	alpha	[0.1, 0.5, 1.0, 2.0, 5.0]	0.1
	l1_ratio	[0.1, 0.3, 0.5, 0.7, 0.9]	0.9
Support vector regression			
	C	[0.1, 1, 10, 100]	10.0
	epsilon	[0.01, 0.1, 1]	1.00
	kernel	['linear’, 'rbf’, 'poly’]	linear

## Results

### The SVR Machine Learning Model Effectively Predicts PSI Scores From the Serious Game Performance Outcomes of Children Diagnosed With ADHD

The predictive performance of the 4 machine learning models was compared to determine which was most effective in predicting the PSI scores of children diagnosed with ADHD from their Neuro-World content performance outcomes (Table 5). SVR demonstrated the best overall performance on the training set, achieving a root mean squared error (RMSE) of 13.107, mean absolute error (MAE) of 9.933, and mean absolute percent error (MAPE) of 9.792% (Table 5). The Elastic Net model exhibited the second highest performance on the training set, with an RMSE of 13.458, MAE of 10.580, and MAPE of 10.352% (Table 5). Random forest and AdaBoost had higher errors on the training set, with RMSE values of 14.307 and 14.269, respectively.

**Table 5.** Comparison of machine learning model performance for predicting processing speed index (PSI) scores of children diagnosed with attention-deficit/hyperactivity disorder (ADHD).

	Training set			Test set		
Model	RMSE^a^	MAE^b^	MAPE^c^ (%)	RMSE	MAE	MAPE (%)
Random forest	14.307	11.813	11.828	15.517	13.165	12.314
AdaBoost	14.269	11.676	11.738	14.511	12.092	11.324
Elastic Net	13.458	10.58	10.352	12.488	8.328	7.993
SVR^d^	13.107	9.933	9.792	11.288	7.874	7.375

a RMSE: root mean squared error.

b MAE: mean absolute error.

c MAPE: mean absolute percentage error.

d SVR : support vector regression.

We then investigated the performance of each model on the test set and found that SVR continued to outperform the other models, with an RMSE of 11.288, MAE of 7.874, and MAPE of 7.375% (Table 5). As with the training set, Elastic Net was second, with an RMSE of 12.488, MAE of 8.328, and MAPE of 7.993%, demonstrating the ability of this model to provide accurate predictions (Table 5). Random forest and AdaBoost again displayed weaker performance. Random forest had an RMSE of 15.517, MAE of 13.165, and MAPE of 12.314%, while AdaBoost had an RMSE of 14.511, MAE of 12.092, and MAPE of 11.324% (Table 4). Overall, SVR emerged as the most effective model for predicting PSI across both the training and test sets.

**Figure 3.**
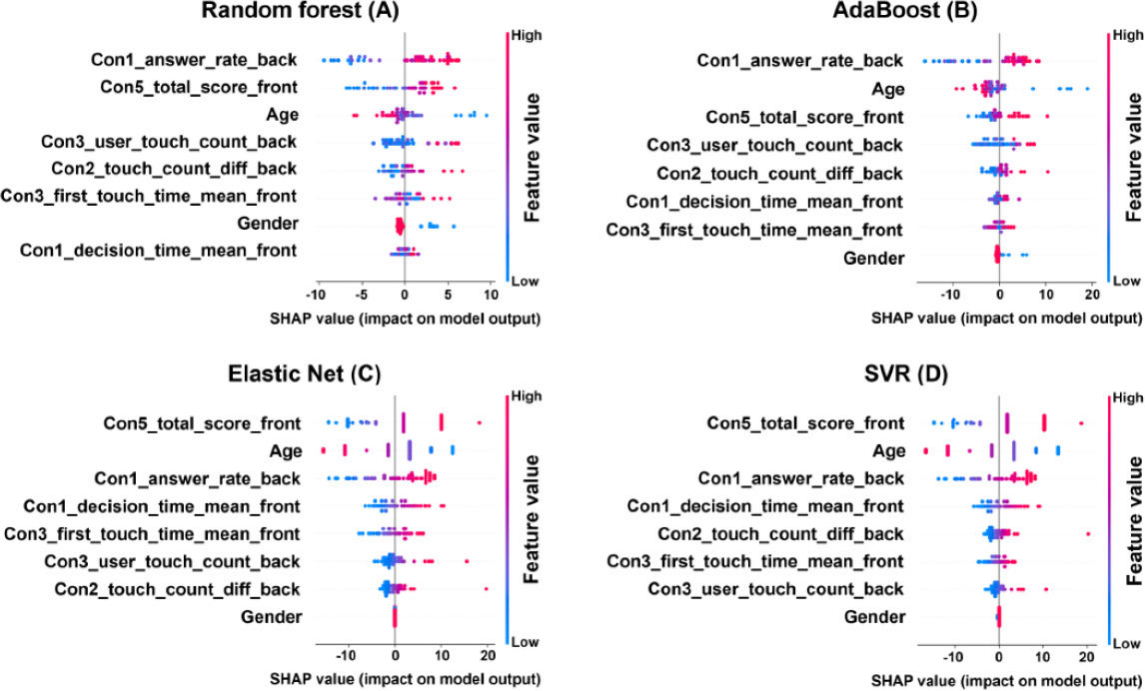
SHAP plot for each machine learning model: (A) Random forest, (B) AdaBoost, (C) Elastic Net, and (D) support vector regression (SVR). Each model represents the top 8 input variables contributing to processing speed index (PSI) prediction. Each dot indicates the SHAP value for an individual participant, and the color indicates the initial value (red: high, blue: low). Features are aligned in order of absolute average SHAP value, and higher values indicate a larger effect of the factor on model output. SHAP: SHapley Additive exPlanations.

To improve the interpretability of the machine learning model, SHAP (SHapley Additive exPlanations) analysis was conducted to assess the contribution of each feature to PSI prediction. Figure 3 presents a summary plot of SHAP analysis results for all machine learning models based on the top 8 input variables for each model (detailed descriptions of these 8 input variables are provided in Table 3). Across all models, “Con5_total_score_front”, “Con1_answer_rate_back”, “age”, and “Con1_decision_time_mean_front” consistently emerged as the main prediction variables. While the relative importance of individual features varied across models, the behavioral performance indices derived from the serious games, including response rate, touch time, and decision time, consistently demonstrated a high degree of predictive efficacy. Gender had a negligible impact on all models. The results of the study support the feasibility of using behavioral performance metrics derived from digital games to predict the PSI scores of children diagnosed with ADHD.

### The Ensemble of AdaBoost and Elastic Net Model Exhibits High Training Performance for Predicting PSI Scores of Children With ADHD

The ensemble technique combines the prediction results of multiple individual machine learning models to achieve superior accuracy and stability performance compared with a single machine learning model [70]. Therefore, to enhance predictive performance by leveraging the strengths of various machine learning models, we applied an ensemble technique using the performance dataset of children with ADHD playing serious game content modules. Specifically, ensemble combinations of random forest, AdaBoost, Elastic Net, and SVR were applied to the training set. The top 5 model combinations with the lowest RMSE scores are presented in Table 6. The combination of AdaBoost and Elastic Net achieved the lowest RMSE of 12.738, indicating the strongest PSI score predictive performance. This combination also demonstrated competitive accuracy, with an MAE of 10.116 and MAPE of 10.086% (Table 6). By contrast, the combination of random forest and Elastic Net had the lowest MAE and MAPE scores of 9.878 and 9.816%, respectively, along with an RMSE of 12.781 (Table 6). These findings showed that combinations of tree-based models (AdaBoost, random forest) with a regression-based model (Elastic Net) outperformed the individual models by leveraging complementary strengths, improving overall predictive performance.

**Table 6.** Top 5 ensemble model combinations ranked by RMSE performance on the training set for predicting PSI^a^ scores of children with ADHD^b^.

Combination	Training set		
	RMSE^c^	MAE^d^	MAPE^e^ (%)
[AdaBoost, Elastic Net]	12.738	10.116	10.086
[AdaBoost, Elastic Net, SVR^f^]	12.770	10.013	9.938
[Random forest, Elastic Net]	12.781	9.878	9.816
[Random forest, AdaBoost, Elastic Net, SVR]	12.802	10.135	10.071
[Random forest, Elastic Net, SVR]	12.821	9.930	9.829

a PSI:processing speed index.

b ADHD: attention-deficit/hyperactivity disorder.

c RMSE: root mean squared error.

d MAE: mean absolute error.

e MAPE: mean absolute percentage error.

f SVR: support vector regression.

### The Ensemble of AdaBoost, Elastic Net, and SVR Exhibits High Testing Performance for Predicting PSI Scores of Children With ADHD

The performance of the ensemble machine learning combinations was then tested using the remaining 12 datasets of Neuro-World content performance of children with ADHD. The combination of AdaBoost, Elastic Net, and SVR resulted in the lowest RMSE of 10.072, with MAE and MAPE values of 6.798 and 6.611%, respectively, indicating high performance on the test set (Table 7). The combination of AdaBoost and Elastic Net, which showed the highest training performance for predicting PSI scores, exhibited the second highest RMSE of 10.089, with MAE and MAPE values of 7.126 and 6.946%, respectively (Table 7). By contrast, the combination of random forest and Elastic Net, which performed strongly on the training set and had the lowest MAE of 9.878, exhibited higher error rates on the test set, with an MAE of 7.788. Together, these results suggest that the PSI prediction accuracy of the ensemble model of AdaBoost and Elastic Net, which provided consistent performance on both the training and test sets, was enhanced by the inclusion of SVR when applied to the test set.

**Table 7.** Test set results of the top 5 ensemble model combinations from the training set for predicting PSI^a^ scores of children with ADHD^b^.

Combination	Test set		
	RMSE^c^	MAE^d^	MAPE^e^ (%)
[AdaBoost, Elastic Net]	10.089	7.126	6.946
[AdaBoost, Elastic Net, SVR^f^]	10.072	6.798	6.611
[Random forest, Elastic Net]	10.432	7.788	7.578
[Random forest, AdaBoost, Elastic Net, SVR]	10.384	7.411	7.272
[Random forest, Elastic Net, SVR]	10.203	7.202	7.035

a PSI; processing speed index.

b ADHD: attention-deficit/hyperactivity disorder.

c RMSE: root mean squared error.

d MAE: mean absolute error.

e MAPE: mean absolute percentage error.

f SVR: support vector regression.

### Comparison of the PSI Score Predictions of the Ensemble Model of AdaBoost, Elastic Net, and SVR With Actual PSI Scores

**Figure 4.**
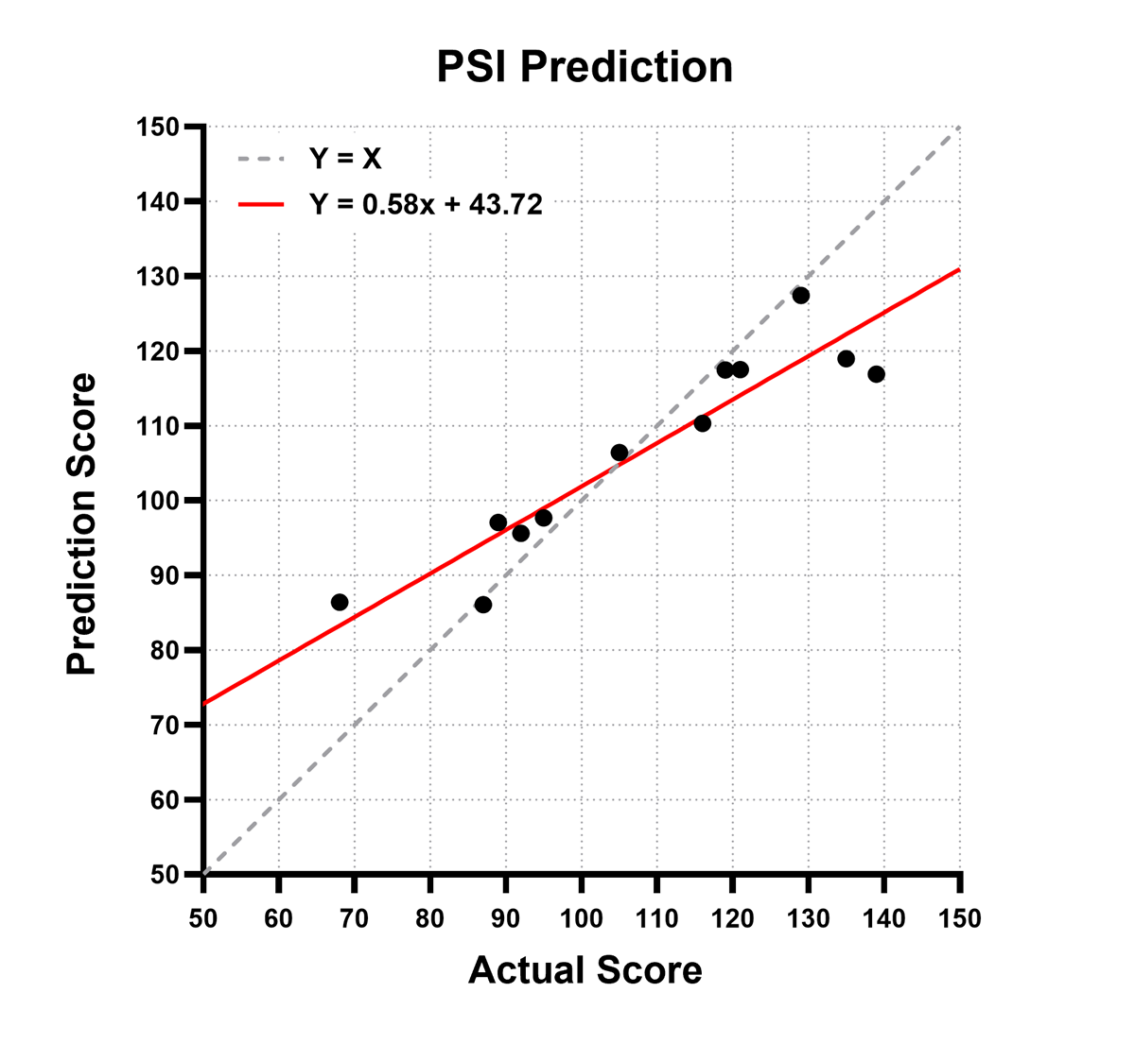
Comparison of actual processing speed index (PSI) values with those predicted by ensemble application. The dashed line (y=x) represents perfect correspondence of the predicted and actual PSI values, and the red line (y=0.58x+43.72) is the regression line of the relationship between the predicted and actual PSI scores.

Since we found that the ensemble model of AdaBoost, Elastic Net, and SVR exhibited high test performance for predicting the PSI scores of children with ADHD, we visualized the prediction scores and actual PSI values on a graph (Figure 4). The x-axis presents the actual PSI scores, while the y-axis presents the scores predicted using the ensemble model of AdaBoost, Elastic Net, and SVR. The data points on the dashed line (y=x) indicate accurate prediction of the actual PSI values. The regression line, expressed as y=0.58x+43.72, reflects the relationship between the predicted and actual PSI scores.

PSI scores typically follow a normal distribution with a mean value of 100, and the intersection of the regression line with the y=x line near a PSI score of 100 suggests higher prediction accuracy for PSI values close to the mean. This alignment highlights the model’s strong performance in accurately predicting PSI scores within the most densely distributed range. Furthermore, most data points closely follow the y=x line, underscoring the model’s reliability and robust predictive capability.

## Discussion

### Principal Findings

Objective methods for tracking ADHD symptoms remain limited in both clinical and nonclinical settings. Due to the lack of standardized measurement tools, symptom monitoring often relies on observation, especially in home and school environments. Although the results of WISC-V assessments are closely linked to daily functioning and thus can be used to quantify symptoms and inform management strategies, such clinical assessments can only be conducted by qualified professionals and cannot be repeated for approximately 2 years. Because traditional clinical assessment tools can only be readministered after a considerable period following the initial psychological evaluation, it is difficult to continuously and objectively monitor symptom improvement. While serious games can be effective means of monitoring progress between hospital visits, most are primarily designed for training and rely on subjective evaluations or intrinsic performance metrics such as task duration and accuracy rates, which have a limited correlation with actual symptom improvement.

**Figure 5.**
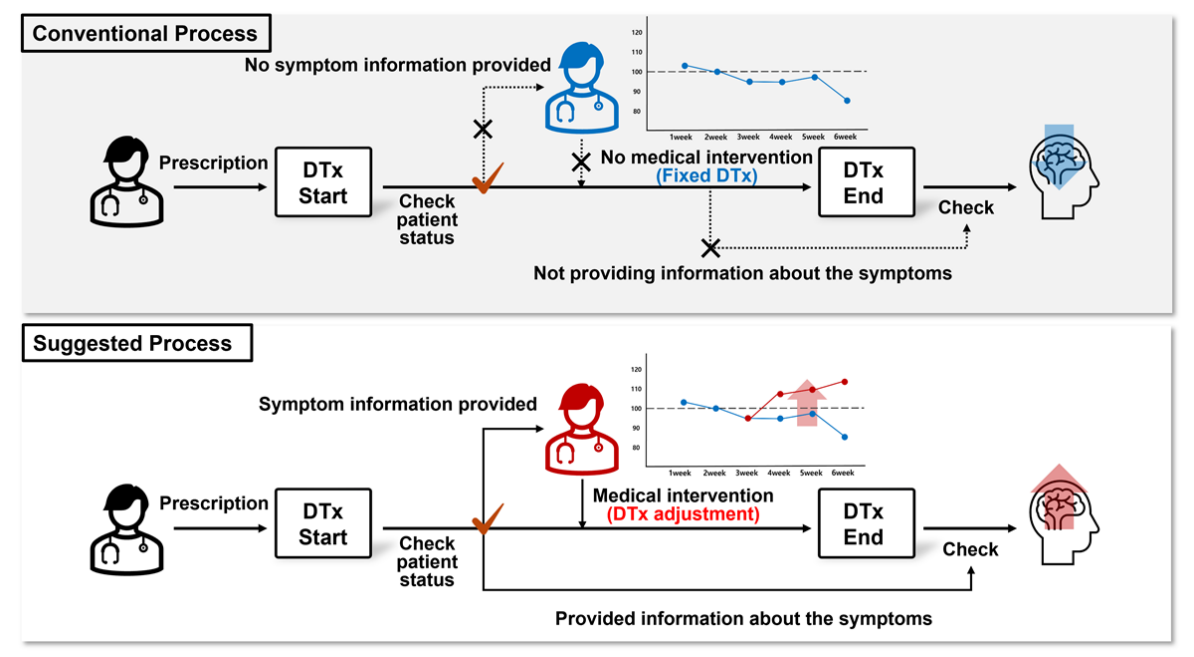
Limitations of traditional digital therapeutics (DTx) monitoring and the need for effective tracking.

To overcome these limitations, this study aimed to integrate serious game performance data with clinically relevant assessment outcomes for ADHD. Specifically, we developed a machine learning-based algorithm to predict PSI scores from behavioral data collected from 59 children aged 6‐13 years diagnosed with ADHD and evaluated the algorithm’s performance. We applied the CFS method to extract 6 variables from the serious game performance data, which were input along with gender and age into machine learning models for analysis. Given the relatively small sample size, we applied cross-validation with varying k values (3, 4, 5, and 6) to enhance the robustness of model evaluation. Among these, k=4 provided the most stable performance without evidence of overfitting. Among the individual machine learning models, SVR was the most effective in predicting the PSI scores of children with ADHD. The combination of AdaBoost and Elastic Net model had the highest training performance for predicting PSI scores, while the ensemble of AdaBoost, Elastic Net, and SVR model had the highest test performance. Ultimately, we developed a model capable of predicting PSI scores using game performance data, enabling estimation of the PSI comparable to that obtained from expert-administered WISC-V assessments (Figure 5).

How can our PSI prediction model be applied in clinical practice or ADHD interventions? The predicted PSI scores can be used to assess symptom improvement using clinically meaningful metrics without time or location constraints. Thus, the model may be feasibly integrated into ADHD care to support ongoing monitoring of cognitive function by complementing or partially replacing standardized assessments such as the full WISC-V, allowing more frequent and accessible tracking of cognitive changes. For instance, regular evaluation of PS using the model may give clinicians continual insights into the treatment responses of pediatric patients with ADHD, enabling timely and precise treatment adjustments. Prior studies have demonstrated the clinical value of machine learning-based predictive models. For instance, a cardiovascular disease risk prediction model developed using machine learning outperformed traditional models in terms of accuracy [71]. Another risk prediction model for cardiovascular disease also demonstrated improved predictive accuracy, particularly in high-risk subgroups such as individuals with diabetes [72]. Machine learning has been shown to enhance both discrimination and calibration in cardiovascular risk prediction, underscoring its potential to improve personalized care in clinical settings [73]. Collectively, these findings support the applicability of machine learning-based PSI prediction for not only diagnosing but also managing and monitoring ADHD.

Our comparison of individual machine learning models revealed that SVR had the highest accuracy for predicting PSI scores, followed by Elastic Net, while random forest and AdaBoost had higher errors on both the training and test sets. Although SVR achieved the highest accuracy in both the training and test evaluations, the 3 other algorithms also exhibited strengths. It is difficult for a single model to achieve sufficient predictive stability and generalizability, and previous studies have shown that leveraging multiple models can substantially improve predictive performance [74]. For example, when multiple models were combined to analyze digital therapeutic log data, accuracy comparisons yielded determination coefficients (R²) ranging from 0.82 to 0.86 [74]. These results support the idea that algorithmic diversity is a key factor in the enhanced efficacy of ensemble methods. When we adopted an ensemble approach in this study, we found that the combination of AdaBoost and Elastic Net outperformed SVR, and the best overall performance was achieved by the ensemble integrating AdaBoost, Elastic Net, and SVR, which was superior to any individual model. These improvements suggest that integrating diverse algorithmic techniques—such as adaptive boosting, regularized linear regression, and complementary model tuning—can significantly improve the reliability of PSI score predictions, a critical factor in ADHD symptom tracking.

The regression line of the PSI prediction accuracy of our model intersected y=x at a PSI score of approximately 100, indicating stable predictive performance in the clinically significant range (approximately 85‐115). This is particularly important for effective monitoring and timely interventions, underscoring the practical applicability of our ensemble model. Similarly, HT Jung et al found that the regression line for MMSE score prediction for MCI crossed y=x near the critical cutoff of 27, indicating high accuracy in distinguishing normal cognition from MCI as well as stability in clinically meaningful ranges [48].

Our findings suggest that the PSI prediction model using serious games can contribute to the quantitative and objective tracking of ADHD symptoms. The ensemble approach effectively compensates for the limitations of individual models, improving predictive stability and accuracy. This method holds promise for clinical use to support continuous patient monitoring and optimization of treatment plans. Furthermore, by integrating this tool into everyday environments such as homes or schools, clinicians may gain valuable insights into symptom tracking and treatment progress beyond those attainable by traditional approaches.

### Limitations

Notwithstanding its contributions, this study has several limitations.

First, this study included 64 participants (59 after excluding incomplete data), a relatively small sample size for developing a generalizable PSI prediction model. Riley et al [75] suggest sample sizes ranging from 100 to several hundred participants to ensure adequate generalizability in clinical machine learning. Although cross-validation was applied to minimize bias, the limited sample size may still restrict reproducibility. In future work, we will validate the generalizability of our prediction model by using a more substantial dataset.

Second, this study focused on pediatric participants aged 6 to 13 years with ADHD, all recruited from South Korea. As the sample was limited geographically and demographically, it is possible that testing of our prediction model on datasets collected from participants from other geographical locations, socioeconomic levels, or backgrounds may yield different outcomes. Although standardized diagnostic procedures were implemented to ensure internal validity, future studies incorporating more diverse populations are needed to improve external validity.

Third, this study used serious game performance data as an alternative to traditional assessment tools. However, repeated use of the serious game may introduce practice effects similar to those observed in the WISC-V assessments. To mitigate practice effects, we used randomization techniques to modulate game complexity in each session. The game uses adaptive mechanisms to adjust difficulty based on individual performance, providing a balanced experience. Nevertheless, there is a paucity of research on the long-term use of game-based assessments and their feasibility. It is imperative to assess the extent of the learning effect and recalibrate the model in the future.

### Future Work

To further enhance our PSI prediction model and facilitate its integration into clinical practice, future studies will use larger and more diverse datasets encompassing varied geographic and demographic populations. Expanding the dataset will also strengthen external validity and improve applicability across broader clinical contexts. In addition, longitudinal studies assessing the long-term reliability of the PSI prediction model by tracking PSI changes over time could further validate its clinical utility in monitoring ADHD-related cognitive function and informing personalized interventions. Finally, incorporating physiological signals, including multimodal data from electroencephalogram, electrocardiogram, and functional near-infrared spectroscopy, may enhance the robustness of the PSI prediction model and advance its potential for medical application in ADHD monitoring and intervention.

### Conclusion

This study introduces a novel approach for predicting K-WISC-V PSI scores using data from serious games designed specifically for children with ADHD. The experimental results showed that among the individual models, SVR had the highest performance, followed by Elastic Net, AdaBoost, and random forest. Furthermore, the ensemble method combining AdaBoost and Elastic Net provided stable and reliable predictive accuracy, highlighting the potential of model integration to enhance overall performance. Our findings suggest that this approach can complement existing subjective methods of treatment monitoring to facilitate continuous symptom tracking and longitudinal monitoring and contribute to the optimization of treatment planning. Integrating PSI prediction with serious game content may enhance patient accessibility and provide a quantitative and reliable tool for clinicians to objectively track and assess a patient’s condition in their everyday environment. Future research should aim to validate these findings using larger datasets including patients of diverse ages and ADHD symptom severities. Incorporating data from participants with varied demographic and clinical characteristics will enhance the model’s generalizability and applicability in real-world clinical environments.

## Abbreviations

ADHD: attention-deficit/hyperactivity disorder
CFS: correlation feature selection
IRB: institutional review board
K-WISC-V: Korean Wechsler Intelligence Scale for Children-Fifth Edition
MAE: mean absolute error
MAPE: mean absolute percentage error
PSI: processing speed index
RMSE: root mean squared error
SVR: support vector regression
WISC: Wechsler Intelligence Scale for Children
