# Correction: A Novel Approach Using Serious Game Data to Predict the WISC-V Processing Speed Index in Children With Attention-Deficit/Hyperactivity Disorder: Machine Learning Study

**DOI:** 10.2196/88500

**Published:** 2025-12-22

**Authors:** Jun-Su Kim, Yoo Joo Jeong, Seung-Jae Kim, Su Jin Jun, Jin-Yeop Park, Hyang-Sook Hoe, Jeong-Heon Song

**Affiliations:** 1 AI-based Neurodevelopmental Diseases Digital Therapeutics Group, Korea Brain Research Institute (KBRI), 61, Cheomdan-ro, Daegu, 41062, Republic of Korea; 2 Neurodegenerative Disease Group, Korea Brain Research Institute (KBRI), Daegu, Republic of Korea; 3 Department of Brain and Cognitive Sciences, Gyeongbuk Institute of Science & Technology, Daegu, Republic of Korea

In “A Novel Approach Using Serious Game Data to Predict the WISC-V Processing Speed Index in Children With Attention-Deficit/Hyperactivity Disorder: Machine Learning Study” [1], the authors noted one error.

In the *Acknowledgments* section, the following information has been added:

JHS and HSH contributed equally as co-corresponding authors on this work, and the latter can be reached at sookhoe72@kbri.re.kr.

The correction will appear in the online version of the paper on the JMIR Publications website, together with the publication of this correction notice. Because this correction was made after submission to PubMed, PubMed Central, and other full-text repositories, the corrected article has also been resubmitted to those repositories.
